# Scaffolds Reinforced by Fibers or Tubes for Tissue Repair

**DOI:** 10.1155/2014/501275

**Published:** 2014-08-18

**Authors:** Xiaoming Li, Nicholas Dunne, Xiaowei Li, Katerina E. Aifantis

**Affiliations:** ^1^Key Laboratory for Biomechanics and Mechanobiology of Ministry of Education, School of Biological Science and Medical Engineering, Beihang University, Beijing 100191, China; ^2^School of Mechanical & Aerospace Engineering, Queen's University of Belfast, Belfast BT7 1NN, Northern Ireland; ^3^Translational Tissue Engineering Center and Whitaker Biomedical Engineering Institute, Johns Hopkins University, Baltimore, MD 21218, USA; ^4^College of Engineering, University of Arizona, Tucson, AZ 85721, USA

The final purpose of tissue engineering is to regenerate or repair defective/damaged tissues and organs. One of vital approaches is to use the various porous scaffolds to simulate the extracellular matrix (ECM) for cellular attachment, proliferation, and differentiation. On the other hand, scaffold materials not only offer mechanical support for embedded cells, but also create a three-dimensional environment for special cells at the same time, which contributes to the repair of tissues and organs. So scaffolds have been an increasingly hot research field because its huge potential in tissue repair engineering [1–3]. Ideal scaffolds are expected to have excellent mechanical properties and biomimetic effect.

In addition to porous and stereo structure, good biocompatibility and biodegradation, and the chance to offer appropriate space environment for cell division and tissue growth, the cracking strength is the essential condition for an ideal scaffold, especially in the condition of tissue implantation [4, 5].

However, generally speaking, considering the mechanical properties of various scaffolds, substantial scaffold materials could not provide enough mechanical support for embedded cells; that is to say, lack of strength restricts the use of scaffolds to great extent. In a sense, the strength of scaffolds determines the scope of its application.

Currently, kinds of fibers or tubes alternatives have been employed to reinforce the scaffolds for repairing specific tissues, such as ceramic fibers/tubes and polymer fibers/tubes [6]. For example, biodegradable synthetic and natural polymers are currently widely used to fabricate tissue engineering scaffolds. Synthetic PLA is a nontoxic, biocompatible, and biodegradable material widely used in tissue engineering [7, 8]. However, the mechanical properties of PLA scaffolds were much lower than that of natural bone. Furthermore, along with the degradation of the scaffold, the overall strength decreases too fast. So, it is adverse for repairing large defect and keeping the restoration for a long time. On the other hand, chitosan is a unique cationic polysaccharide with many attractive properties, including hydrophilicity, nontoxicity, and cell affinity [9–12]. To reinforce PLA scaffold, chitosan fibers are used to be natural reinforced phase; for example, Jiao et al. reported that coating chitosan on a microscale PLA scaffold could improve its mechanical strength and cell compatibility [13]. Similarly, calcium phosphates are commonly used materials for the restoration of bone defects with excellent biocompatibility and bioactivity. However, brittleness and low flexural/tensile strength so far limit their application to nonload bearing areas. Reinforcement of calcium phosphate cements with fibers can substantially improve its strength and toughness and has been one major strategy to overcome the present mechanical limitations of calcium phosphate cements. Fiber reinforced calcium phosphate cements thus bear the potential to facilitate the use of degradable bone substitutes in load bearing applications. There are lots of cases like the above examples; we will not introduce one by one; in one word, although three-dimensional porous scaffolds posses outstanding biocompatibility, biodegradability and also have huge promising application in different tissue regeneration, to meet different needs of clinical applications, mechanical properties of desirable scaffolds should be further improved by means of addition of different appropriate fibers or tubes. However, a new problem emerges simultaneously, for the purpose of reinforcing the scaffold truly, on how we disperse the reinforcing phase into matrix uniformly, thus acquiring a homogeneous structure and component scaffolds.

In most cases, the interaction of fillers and matrix determines the reinforcing effect directly. Some measures could be taken to increase the bonding force between fillers and matrix, such as disperse fibers or tubes well as far as possible and functionalize the surface of fillers in order to enhance the scaffold. What is more, additional studies are required for making a better understanding of how the fibers or tubes influence the strength and biocompatibility of the scaffolds.

In this special issue, several articles are mainly focus on some specific applications of scaffolds reinforced by fibers or tubes, a few novel fibers or tubes with excellent performance, and the interactions between fibers or tubes and matrix as well as mechanisms of reinforcement of fibers or tube. Not only up-to-date reviews and application about scaffolds reinforced by fibers or tubes for tissue repair could be found, but also the impact of fibers or tubes on the biocompatibility and biodegradability of the scaffolds in this issue. Besides, it will make you have a good understanding of the chemical functionalization of different fibers or tubes and different kinds of link force, such as chemical bond, Van der Waals' force, and physical force. All in all, the issue will give a brief presentation about scaffolds reinforced by fibers or tubes, and most importantly, it will provide a general guide for the fabrication of more desirable scaffolds.

*Xiaoming  Li* *Nicholas  Dunne* *Xiaowei  Li* *Katerina E.  Aifantis*
